# Ultraviolet and Infrared Irradiations Sensing of Gel-Orange Dye Composite-Based Flexible Electrochemical Cells

**DOI:** 10.3390/gels8020083

**Published:** 2022-01-28

**Authors:** Muhammad Tariq Saeed Chani, Khasan S. Karimov, Abdullah M. Asiri, Tahseen Kamal, Esraa M. Bakhsh, Mohammed Muzibur Rahman

**Affiliations:** 1 Center of Excellence for Advanced Materials Research, King Abdulaziz University, P.O. Box 80203, Jeddah 21589, Saudi Arabia; aasiri2@kau.edu.sa (A.M.A.); tkkhan@kau.edu.sa (T.K.); mmrahman@kau.edu.sa (M.M.R.); 2 Chemistry Department, Faculty of Science, King Abdulaziz University, P.O. Box 80203, Jeddah 21589, Saudi Arabia; ibakhsh@kau.edu.sa; 3 Ghulam Ishaq Khan Institute of Engineering Sciences and Technology, Topi 23640, Pakistan; khasan@giki.edu.pk; 4 Center for Innovative Development of Science and Technologies of Academy of Sciences, Rudaki Ave. 33, Dushanbe 734025, Tajikistan

**Keywords:** electrochemical cell, gel electronics, ultraviolet and infrared irradiation sensing, organic semiconductor, flexible and shockproof devices, impedance

## Abstract

The flexible and shockproof rubber-based Al/OD-Gel/Cu electrochemical cell was designed, fabricated, and investigated for the detection of IR and UV irradiations. For this purpose, the transparent gel–orange dye composite was deposited on the porous rubber substrate between aluminum and copper electrodes. It was observed that the gel–orange dye composite was mechanically like a gel: soft and flexible. Electrically, this composite (gel–orange dye) forms a flexible electrolyte. It was found that the impedance of the samples under the effect of infrared irradiation decreased by 2.02 to 2.19 times on changing frequency from 100 Hz to 200 kHz. Accordingly, under the effect of ultraviolet irradiation, the impedance of the samples decreased by 1.23 to 1.45 times on increasing frequency from 100 Hz to 200 kHz. Under the effect of infrared irradiation up to 4000 W/m^2^, the cell’s open-circuit voltage increased by 1.59 times. The cell’s open-circuit voltage also increased by 1.06 times under the effect of ultraviolet irradiation up to 200 uW/cm^2^. The mechanism of the absorption of the infrared and ultraviolet irradiations by the OD–Gel composite has been discussed in detail. The fabricated flexible rubber substrate-based Al/OD-Gel/Cu electrochemical cells can be used as a prototype for the development of gel electronics-based devices.

## 1. Introduction

Electrochemical, infrared, and ultraviolet sensors are used in different areas of modern technologies [1,2]. At the same time, the combinations of these sensors may be considered as a new stream in sensor and electrochemical devices technologies at the present time. Electrochemical sensors based on organic conjugated polymers were discussed in ref. [3]. The films of a nanocrystalline metal oxide containing adsorbed perylenediimides’ derivative (polyether) were studied for electrochemical and optical properties [4]. In ref. [5], graphene-based electrochemical sensors and biosensors were reviewed. All-organic semiconductors for the electrochemical biosensors: an overview of recent progress in materials design was presented in ref. [6]. During the last years, paper-based electronics were introduced, as well: electrochemical sensors using paper as a scaffold to create porous carbon nanotube electrodes was published [7]. Newly designed, fabricated, and investigated infrared detectors were discussed in ref. [8]. These detectors were mainly comprised of an emitter region, a collector region, a pair of contacts, and a barrier region. The barrier and emitter were made of ternary alloys of the same species, such as n/n, n/p or p/n-type compositions of indium gallium arsenide, cadmium mercury telluride, or gallium aluminum arsenide. A semiconductor film bolometer thermal infrared detector was patented, as well [9]. The detector was supported by a pellicle (aluminum oxide/polyamide) thin film suspended in the cavity of the substrate (monocrystalline silicon wafer). The detector itself was comprised of heat-sensitive semiconductive (amorphous silicon) film sandwiched between two metallic (platinum/tantalum) electrodes. The chitin based organic semiconducting films making infrared systems and devices [10] and detection of chemicals with infrared light [11] were described in the concerned patents. Graphene-based wearable infrared photodetector and temperature sensors fabricated on polyimide flexible substrates were presented in ref. [12]. The organic photodiodes and phototransistors were fabricated and investigated for infrared detection [13]. In ref. [14], the ultraviolet light sensors based on facile constructive heterojunction of ZnO nanorod/PEDOT:PSS were fabricated and discussed with their mechanism and efficient performance.

Along with infrared detectors, due to the demands of the developing technologies and industries, much attention was paid to the fabrication and investigation of ultraviolet sensors, as well. The blends of naphthalenediinaide and polyfluorene were used to fabricate highly proficient UV photodetectors, and the effect of thermal annealing on their performance was studied in detail [15].

The effect of ultraviolet radiation on organic photovoltaic materials and devices was described in ref. [16]. Ferroelectric infrared sensors and methods for their manufacturing were presented in ref. [17]. The sensing structure was mainly based on platinum electrodes and a ferroelectric (strontium bismuth tantalate) layer. A soft biocompatible material-based piezoelectric nanogenerator as a non-disposable and reusable energy harvester was fabricated, which can be used by enclosing it in the mask [18]. These nanogenerators are mainly based on electrospun membranes of pure PVDF poly(vinylidene fluoride) and PVDF with cardanol oil on a flexible laser-ablated polyimide substrate. The substrate was attached with a blend of Ecoflex and poly(dimethylsiloxane) (PDMS) as an elastomeric skin-comforter. In addition to having polymeric properties, the PVDF exhibits a signal voltage similar to lithium single crystal [19]. A self-supporting PVDF film, having four elemental arrays, was designed to detect human body motion. Such an array may be used for area supervision and traffic monitoring by the evaluation of directional motion [19]. For the proficient ultraviolet photodetection using an organic semiconductor/silicon (hybrid) photodiode was presented in ref. [20]. In ref. [21], the display with an infrared backlight source and multi-touch sensing function was described. The wide bandgap semiconductor nanowires based solid-state UV detectors were reviewed in ref. [22].

The detection of infrared and ultraviolet radiations and related device manufacturing techniques were described in ref. [23]. The properties of the bolometers, having an organic semiconductor layer arrangement, were presented in ref. [24].

In ref. [25], the infrared sensor arrays and photodiodes with enhanced passivation layers and manufacturing methods were described. In ref. [26], the diode bolometer and method for producing a diode bolometer were described.

Presented references show that for fabrication of the organic materials-based infrared sensors, mostly polymers, graphene, and carbon nanotubes were used by utilization of the well-known traditional technologies. At the same time, in some particular cases, for example, ultraviolet sensors, not only organic polymers but inorganic materials were used as well, for example, ZnO.

Analysis of published papers and patents showed that these publications presented information about the composition, structure, and properties of the IR and UV sensors fabricated up to the present time, on the basis of organic and inorganic materials. At the same time, practically, less information about the organic semiconductor-based devices firstly and secondly about the combined infrared and ultraviolet sensors. This approach could allow us to widen application areas, decrease the total cost of the devices, and finally, decrease the negative experimental effect at the fabrication of the devices. In this regard, the flexible and shockproof rubber-based Al/OD-Gel/Cu electrochemical cells were designed, fabricated, and investigated for the detection of IR and UV irradiations. For this purpose, environmentally friendly rubbing-in technology was used to fabricate the cells. The fabricated flexible electrochemical cells have potential use as a prototype for the development of gel electronics.

## 2. Results and Discussion

**Figure 1:**
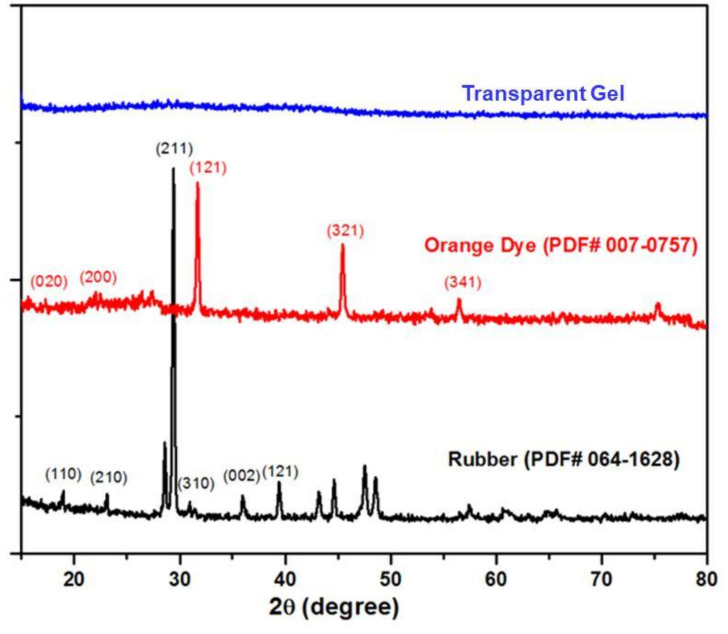
XRD scans of the OD, transparent gel, and rubber substrate.

XRD scans of the OD, transparent gel, and rubber substrate are shown in Figure 1. The samples were scanned using Philips PW1830 XRD in θ–2θ scan mode under a Cu-Kα (monochromatic) radiation source with 40 kV accelerating voltage and 25 mA tube current at 25 °C. The step size was 0.05° during scanning between 2θ angles 15° and 80°. The XRD scans for each sample (rubber, orange dye, and transparent gel) were repeated three times. The rubber shows (Figure 1) high intensity peaks at the angles (2θ) of 18.86°, 23.30°, 29.73°, 30.87°, 36.23°, and 39.29°; their corresponding miller indices are (110), (210), (211), (310), (002), and (121), respectively. All the peaks shown in the rubber’s XRD pattern depict the polyvinyl chloride’s characteristics that matched with PDF # 00-064-1628 of the ICDD database. These peaks correspond to the high structural order of polymeric chains. The orange dye (OD) compound is ammonium oxalate hydrate (C_2_H_8_N_2_O·H_2_O), and peaks are matched with the standard XRD database (PDF# 00-007-0757) and consistent with previous studies. No diffraction peak was observed in the X-ray diffractogram of the transparent gel.

**Figure 2:**
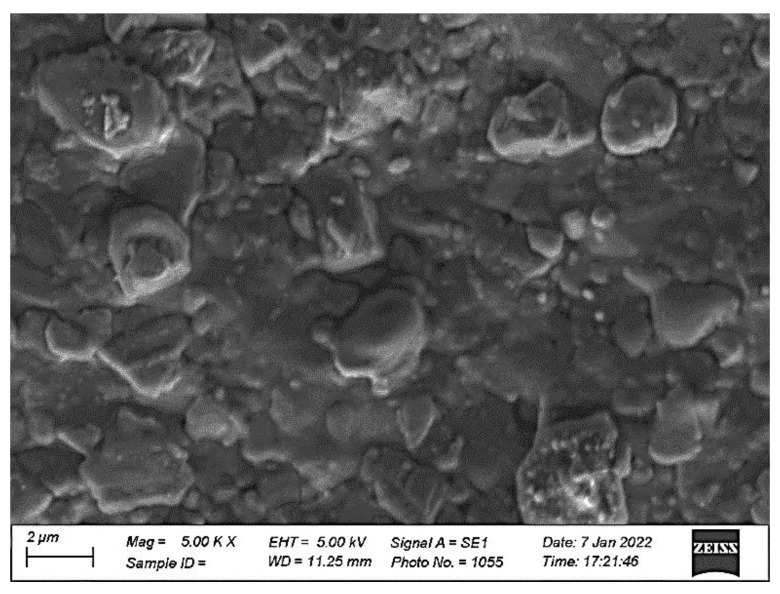
The SEM image showing the surface morphology of the orange dye film. The surface roughness and porosity are more pronounced in the micrograph.

Figure 2 shows the surface morphology of the orange dye film deposited on a glass substrate. The surface roughness is more pronounced in the micrograph. The microstructure also shows a high level of porosity. The surface roughness and higher porosity provide a high active surface area, which is required for the devices to enhance the sensitivity.

**Figure 3:**
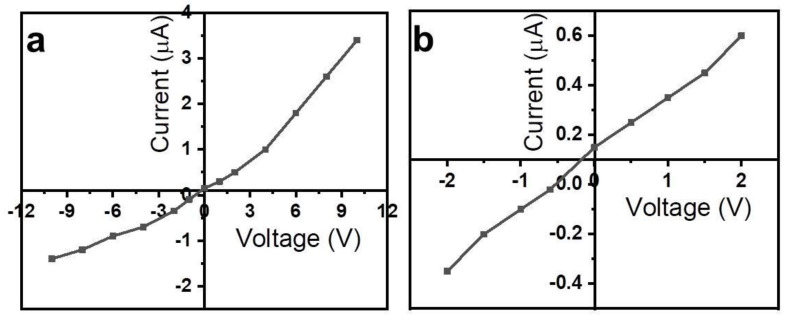
Volt-ampere characteristics of the rubber-based Al/OD-Gel/Cu electrochemical cells in the wide range of applied voltages (**a**) and narrow range of applied voltage (**b**).

Figure 3a,b shows the volt-ampere characteristics of the Al/OD-Gel/Cu electrochemical gel cells in wider and narrow ranges of the applied voltages, respectively. It is seen that I-V characteristics show the rectification behavior with the ratio equal to 2.4 and 1.7, accordingly. The origin of the rectification behavior is probably considered the difference of electrochemical potentials of the Al (−1.66 V) and Cu (+0.34) electrodes, which influences as differences of the forward and reverse biases, as in semiconductors and metal-semiconductor (Schottky junction) rectifiers.

**Figure 4:**
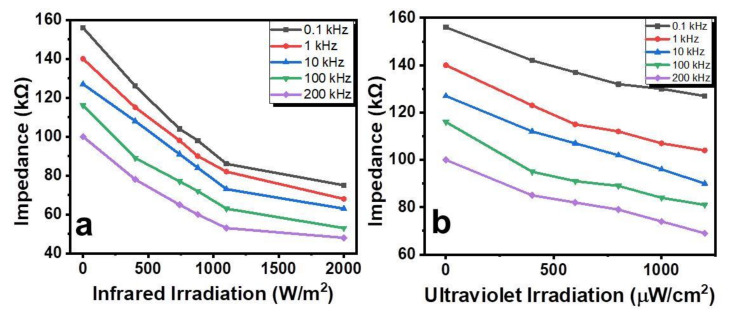
Dependence of the Al/OD-Gel/Cu cells impedance on different frequencies under the infrared (**a**) and the ultraviolet (**b**) irradiation.

Figure 4a shows that the impedance of the cells decreased under the effect of infrared irradiation. This decrease in impedance was in the range of 2.02 to 2.19 times on changing frequency from 100 Hz to 200 kHz. The initial impedance of the sensors decreased by increasing the frequency. In response to the frequency changing from 100 Hz to 200 kHz, the initial impedance decreased from 156 kΩ to 100 kΩ. The impedance-ultraviolet irradiation behavior of the sensors is shown in Figure 4b. It can be seen that the impedance of the cells decreased under the effect of ultraviolet irradiation. The impedance decreased on average by 1.23 to 1.45 times in response to a frequency change from 100 Hz to 200 kHz.

**Figure 5:**
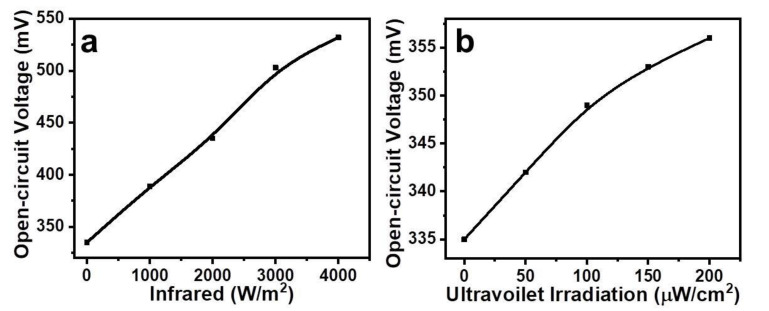
Open-circuit voltage-infrared irradiation (**a**) and open-circuit voltage-ultraviolet irradiation (**b**) relationships of Al/OD-Gel/Cu cells.

The open-circuit voltage-infrared irradiation relationship is shown in Figure 5a. On increasing infrared irradiation from 0 to 4000 W/m^2^, the open-circuit voltage of the cells increased by 60%. Similarly, the open-circuit voltage-ultraviolet irradiation relationship is shown in Figure 5b. Under the effect of the ultraviolet irradiation from 0 to 200 µW/cm^2^, the cells’ open-circuit voltage was augmented by 10%. The origin of these effects can be explained, firstly, by the changes in the concentration and mobility of the organic semiconductor orange dye-gel composite. Moreover, probably, the contribution of the contact resistances of Al/OD-Gel and Cu/OD-Gel metal-semiconductor junctions takes place as well, which may be originally ohmic or rectifying contact by nature.

The experimental results which were presented in Figure 4 and Figure 5 can be explained briefly by the thermal effect of the infrared and ultraviolet irradiations on the concentration and mobility of the charges in the OD–gel composite and, to some extent, on the electrode potentials between Al/OD-Gel and Cu/OD-Gel, as well. More detailed investigations are required for confirmation of these suppositions.

**Figure 6:**
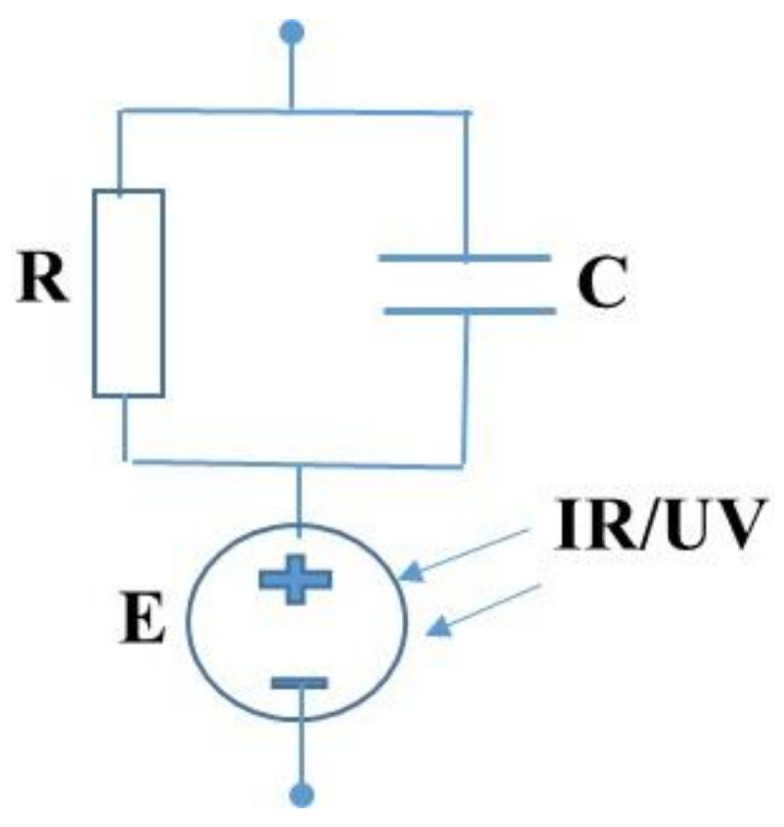
Simplified equivalent circuit of the Al/OD-Gel/Cu electrochemical cell which contains parallel connection of the resistance (R) and capacitance (C) with voltage source connected in series.

The equivalent circuit of the Al/OD-Gel/Cu electrochemical cell is shown in Figure 6, where the resistance (*R*) and the capacitance (*C*) are connected in parallel, while the voltage source (*E*) is connected in series. The fabricated electrochemical sensors have two metallic electrodes with different standard electrochemical potentials. That is why the generation of the electric voltage takes place.

**Figure 7:**
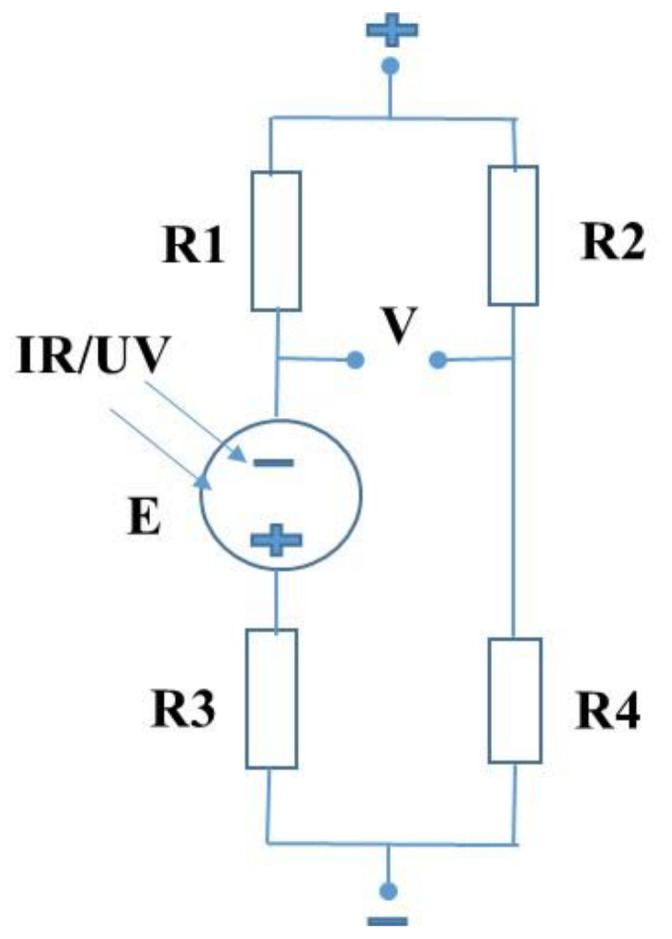
Wheatstone bridge-based circuit for the measurement of the IR and UV lights intensities by use of the Al/OD-Gel/Cu electrochemical Cell (E and R3).

In continuation of this work, on the basis of the well-known Wheatstone bridge, it was designed, fabricated, and tested in the circuit shown in Figure 7. This circuit allowed us to measure the intensity of infrared and ultraviolet irradiations and the voltage generated by the cell. The proper selection of the polarities of the voltage source to the Wheatstone bridge and terminals of the Al/OD-Gel/Cu electrochemical cell allowed us to increase the sensitivity of the circuit at the measurement of the intensities of the *IR* and *UV* irradiations. A comparison of fabricated sensors with the sensors reported in the literature is given in Table 1. This comparison is on the basis of materials, fabrication techniques, and sensing ranges. From Table 1, it can be elucidated that the gel-based sensors are promising in terms of low cost, environmentally friendly fabrication technique, and performance.

## 3. Conclusions

Infrared and ultraviolet irradiation sensors based on orange dye-gel composite flexible electrochemical cells were fabricated by simple, economical, and environmentally friendly rubbing-in technology. For the fabrication of cells, commercially available materials, such as pristine orange dye, gel, and elastic rubber substrates, were used. The sensor showed multiple-sensitivity to infrared irradiation (i), ultraviolet irradiation (ii), polarity of the applied voltage (iii), Wheatstone bridge and connection of terminals (iv), and the sensor generated voltage (v), as well. The cell’s impedance decreased under the effect of infrared and ultraviolet irradiations by 2.02–2.19 and 1.23–1.45 times, respectively, on changing the frequency from 100 Hz to 200 kHz. Under the effect of infrared irradiation up to 4000 W/m^2^, the cell’s open-circuit voltage increased by 1.59 times. The sensor, which was a built-in Wheatstone bridge, was connected in such a way that the polarities of the voltage dropped on the sensor due to the voltage supply of the bridge. The polarity of the voltage supply to the bridge would be the opposite of the polarity of the voltage generated by the electrochemical cell. This opposite polarity caused an increase in the sensitivity of the infrared and ultraviolet irradiations sensors based on the flexible electrochemical cells. The Al/OD-Gel/Cu rubber composite electrochemical cells fabrication and utilization can be used as a prototype for the development of gel electronic-based devices, which may be shockproof and shake-proof.

## 4. Experimental

**Figure 8:**
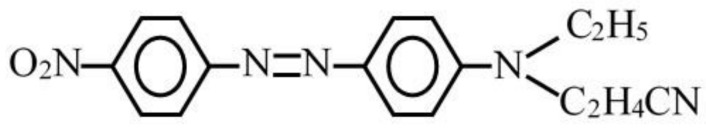
Molecular structures of orange dye (OD).

For the fabrication of electrochemical cells, the orange dye (OD), having 95% dye content, was purchased from Sigma Aldrich (https://www.sigmaaldrich.com/SA/en/product/aldrich/364819, accessed on: 20 November 2021) (Merck, KGaA, Darmstadt, Germany). The orange dye (C17H17N5O2) is an organic semiconductor material having a density of 0.9 g/cm^3^ and a molecular weight of 323.35 g/mol. The OD’s IUPAC name is 3-[N-Ethyl-4-(4-nitrophenylazo)phenylamino]propionitrile, and it has p-type conduction. The molecular structure of orange dye is shown in Figure 8. Being an organic semiconductor, the orange dye (C_17_H_17_N_5_O_2_) has an energy gap in the energy spectrum similar to silicone. Therefore, this type of orange dye is attractive for infrared and ultraviolet irradiation sensing. The transparent gel used in these cells is made of cross-linked sodium polyacrylate with 70 wt.% water. The gel was also purchased from the market (Tianhe District, Guangzhou, China) (http://www.miracle-chemical.com/Products.asp?ClassID=115, accessed on: 20 November 2021). The substrate selected for the ultraviolet and infrared irradiation sensors was elastic rubber.

**Figure 9:**
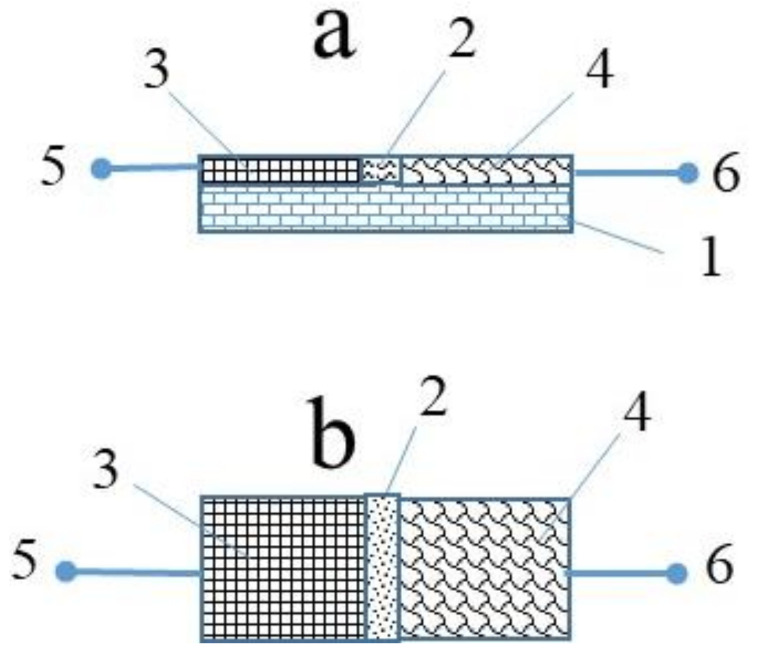
Schematic of the front view (**a**) and the top view (**b**) of the rubber based Al/OD-Gel/Cu composite flexible electrochemical cells: rubber substrate (1), OD–gel composite (2), Al electrode (3), Cu electrode (4), metallic terminals (5 and 6).

To fabricate the sensors, first of all, the OD-gel adhesive composite was prepared by mixing OD and gel with the proportion of 40 wt.% and 60 wt.%, respectively. To fabricate the Al/OD-Gel/Cu shockproof and flexible electrochemical cells, the rubber was used as a substrate. The sizes of the rubber substrate were the following: length, width, and thickness were equal to 20 × 10 × 10 mm^3^. To form an active layer, the already prepared OD–gel composite was deposited between aluminum (Al) and copper (Cu) foil electrodes using rubbing-in technology. The Al and Cu foil electrodes were already fixed on the surface of rubber substrates by conducting paste (silver paste or aquadag). The gap between the two electrodes was equal to 2–3 mm, and the electrodes were in electrical contact with the built-in OD–gel composite layer. The thickness of the OD–gel composite layers was in the range of 17–21 μm. The schematic diagram of the fabricated Al/OD-Gel/Cu shockproof and flexible electrochemical cell is shown in Figure 9.

For the measurements of the impedance in the frequency range of 100 Hz to 200 kHz, the digital LCR meter MT 4090 was used, while the DT 4253 digital multimeter was used for the measurements of voltage. The intensities of the infrared and ultraviolet irradiations were measured by LS122 IR power meter (Linshang Technology, Shenzhen, China) and UV light meter UV-340A (Lutron Electronics, Coopersburg, PA, USA), accordingly. The thickness of the OD–Gel composite layers was measured by an optical microscope with a built-in micrometer scale. The fabrication of the sensors was simple and did not require complicated and expensive equipment to allow us to fabricate the devices not only for research purposes but for teaching purposes in education laboratories, as well.

## Figures and Tables

**Table 1:** Comparison of fabricated sensors with the sensors reported in the literature.

Sr. No	Sensor Type	Materials Used	Fabrication Technique	Sensing Range	Impedance/Resistance Change	Ref.
1	UV	ITO/PEDOT: PSS/PFE:BNDI	Spin coating	0–1 mW/cm^2^	61.8 to 19.5 kΩ	[15]
2	UV	CH3NH3PbI3-xClx perovskite	Spin coating	0–200 W/m^2^	7.4 to 2.0 MΩ	[27]
3	UV	ZnO	Hand spread	0–1.6 mW/cm^2^	2.6 to 0.7 MΩ	[28]
4	UV	gel–orange dye	Rubbing-in	0–1200 μ W/cm^2^	127 to 90 kΩ	Present work
5	IR	CH3NH3PbI3-xClx perovskite	Spin coating	0–6000 W/m^2^	9.0 to 5.0 kΩ	[27]
6	IR	Micromachined piezoelectric resonator	MEMS	0–1.0 mW/mm^2^	2.7 to 1.0 GΩ	[29]
7	IR	gel–orange dye	Rubbing-in	0–2000 W/m^2^	156 to 75 kΩ	Present work

PFE: Poly(9,9-dioctyl fluorenyl-2,7-yleneethynylene); BNDI: N,N-bis-n-butyl-1,4,5,8-naphthalenediimide.
